# Volatile Organic Compounds (VOCs) from Wood and Wood-Based Panels: Methods for Evaluation, Potential Health Risks, and Mitigation

**DOI:** 10.3390/polym12102289

**Published:** 2020-10-06

**Authors:** Tereza Adamová, Jaromír Hradecký, Miloš Pánek

**Affiliations:** Faculty of Forestry and Wood Sciences, Czech University of Life Sciences, Kamýcká 129, 165 00 Prague 6, Czech Republic; hradecky@fld.czu.cz (J.H.); panekmilos@fld.czu.cz (M.P.)

**Keywords:** wood, wood-based panels, volatile organic compounds (VOCs), indoor air, gas chromatography-mass spectrometry (GC-MS)

## Abstract

Volatile organic compounds (VOCs) are contained in various construction materials and interior equipment. Their higher concentrations in the indoor air are associated with negative effects on human health and are disputed in terms of health risk, since people spend a considerable part of their lifetime indoors. Therefore, the presence of VOCs in indoor air is a case of concern regarding sick building syndrome (SBS). From a historical point of view, wood and wood-based panels represent a widely used material. Nevertheless, wood appears to be nowadays a product and a material of a sustainable future. Depending on wood extractives’ composition and an abundance of diverse wood species, different profiles of volatiles are emitted. In case of wood-based panels, the impact of adhesives and additives that are essentially applied aiming to adjust the panels’ properties is even enriching this cocktail of chemicals. This paper comprises the issue of VOCs emitted from wood and wood-based panels. The most abundant VOCs were summarized. The options of VOCs for analytical determination from these matrixes are described with their benefits and limitations.

## 1. Introduction

Volatile organic compounds (VOCs) are a large group of various compounds including natural compounds as terpenes, alcohols, but also carbonyl compounds as ketones, aldehydes, ethers, aromatic hydrocarbons, and acids, which are the main pollutants present in indoor air [1,2]. As described by the World Health Organization [3], VOCs are, besides semivolatile organic compounds (SVOCs) and very volatile organic compounds (VVOCs), any organic compound with a boiling point in the range of 50–100 °C to 240–260 °C. VOCs sources are divided into two groups—natural or anthropogenic. The natural sources are mainly represented by green vegetation, which is an emission source that cannot be actually controlled. Thus, human activities, such as manufacturing, petrochemical refinement, and vehicle emissions represent anthropogenic sources [4,5,6]. Some VOCs such as formaldehyde are both from natural and anthropogenic origin. In some regional areas, the emissions of VOCs generated by human activities proved to be much higher than those from natural sources [7]. Nevertheless, due to control and emission mitigation programs, the anthropogenic VOCs emissions are likely to decrease in the future, and the significance of biogenic VOCs may become more important [8]. Since VOCs are considered gaseous pollutants that can be brought in or infiltrate from outdoor to indoor environments, indoor air quality (IAQ) and its pollution is an issue in developed countries. Therefore, an indoor/outdoor ratio was established to evaluate the grade of VOCs infiltration in urban areas, as indoor air pollution became a main determinant of human respiratory health [9,10]. Many types of VOCs are photochemically sensitive; ozone and other hazardous products are formed when exposed to nitrogen oxides and sunlight [5,11,12], and several VOCs were considered respiratory toxic [13]. As VOCs concentrations measured indoors typically exceed those outdoors [14], it is crucial to keep in mind the potential health risk consequences of indoor exposure to VOCs [15,16], since people in developed countries in the 21st century spend a considerable part (approximately 90%) of their lifetime indoors. Additionally, in certain conditions, inhabitants of poorly ventilated buildings are more prone to suffer from “sick building syndrome” (SBS) [17], which is a phenomenon characterized by various symptoms such as headache; eye, nose, or throat irritations; dry cough; allergy reactions; dry and itching skin; nonspecific hypersensitivity; insomnia; dizziness and nausea or difficulty in concentrating; and tiredness [18]. The intense odors may have a negative psychological influence as well [19]. Moreover, Singleton et al. [20] describe the vulnerability of the liver, Jain [21] links humans’ exposure to VOCs with kidneys regression, and the study of Cakmak et al. [22] brings out the harmful effect of VOCs exposure on male and female lungs function.

**Figure 1:**
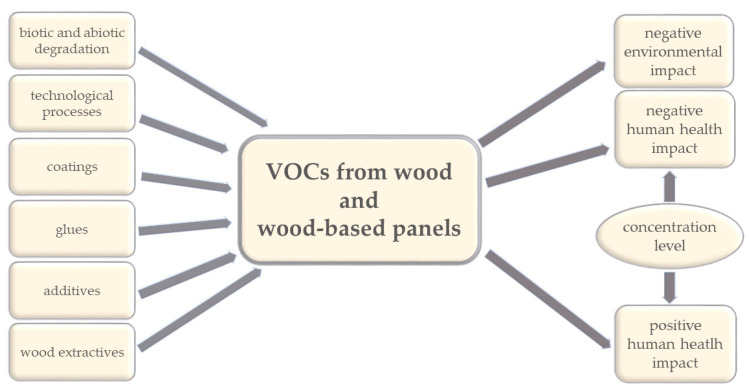
Volatile organic compounds (VOCs) from wood and wood-based panels: their sources and impact.

In interiors, VOCs are primarily emitted from indoor sources such as building materials, parquets, particle boards, oriented strand boards, plywood, furniture containing formaldehyde-based resins [2,23,24,25] from finishes, including surface materials such as polyvinyl chloride (PVC)/vinyl or linoleum, glues, paints, and floor coverings (Figure 1), and from consumer products such as cleaning products, personal care products, fragrances, and air fresheners [1,26,27,28,29]. The results reported by Ewen [30] indicate that wood-rotting fungi may be also a contributory factor in “sick building syndrome”, since most houses could be expected to contain VOCs emitted from fungi from various parts of a building (e.g., from behind paneling or skirting boards). Some of the VOCs identified from wood-rotting fungi have particularly potent odors, and some of them represent a possible health risk. Therefore, the thorough selection of building materials plays a key role in its occupants’ health state [31].

In addition, VOCs release depends on the prevailing thermal and moisture conditions, the air pressure difference over the structure, the structural design and the quality of the construction work, the volume of air contained in the indoor space, the rate of production or release of the volatile compound, the rate of removal of the pollutant from the air via reaction or settling, and the rate of air exchange with the outside atmosphere [32,33].

Considering instrumental methods used to determine the VOCs, gas chromatography–mass spectrometry (GC-MS) is commonly used to separate and identify the volatiles. For formaldehyde determination, liquid or gas chromatography is used, often after derivatization. Regarding volatiles extraction, exhaustive extraction techniques can be used for compounds concentration evaluation in solid material, while equilibrium techniques are used to monitor compounds abundances in a defined space of air to describe their emission from solid material or to monitor indoor air quality.

## 2. VOCs from Wood

Wood is a common natural product with a typical pleasant smell composed of main structural compounds of polysaccharides (cellulose, hemicelluloses, and lignin) that contain a wide range of low molecular weight organic chemicals and extractives [34,35,36]. Their content varies from 0.5 to 20 weight (wt) % [37] and can be readily extracted from wood with neutral organic solvents or water. It is well known that the content of wood extractives correlates closely with the quality of wood [38,39]. Extractives often are of decisive importance in contributing to many of the characteristic properties and possible uses of wood, such as its odor, color, light stability, flammability, hygroscopicity, density, strength properties, decay, insect resistance, and permeability [40]. According to the extraction method, the wooden extractives can be divided into groups—lipophilic or hydrophilic (or polar) components [41,42]. An important portion of wood extractives are volatile organic compounds (VOCs) formed by terpenes, terpenoids, flavonoids, alcohols, aldehydes, and ketones, also in smaller amounts of higher alkenes and fatty acids [43]. This is a low, but still well detectable, amount of VOCs that can be released from wood [44]. The presence of terpenes in wood is primarily linked to the resin. In the sapwood of conifers and deciduous trees, the resin flows in parenchyma cells and resin canals. In parenchyma cells, it consists of terpenes, esters, fats, and waxes; in resin canals, it is composed of resin acids and volatile terpenes. The heartwood of conifers contains most of the terpenes in resin canals [45]. For example, in pine, resin acids represent 67% of extractives’ content, while in spruce, they do not exceed 24% [46]. Mono-, di-, and sesquiterpenes are the dominant VOCs for conifers, while triterpenes and sterols are predominant in deciduous trees.

Extractives of certain kinds of wood are used in many medical products and in the perfume industry. Their impact on human health can be negative [44], but also positive [47], as disputed in the study of Pei and Yin [48], who consider new furniture and wood-based decorations to be gas pollutant sources that affect the conditions in indoor environments. In contrast, the study from Xi et al. [49] highlights the benefits of a wooden indoor environment to its occupants who suffer less tension and fatigue, as VOCs emitted from wood can have a positive effect, especially on the nervous, respiratory, and visual system.

The content and type of extractive substances that can be released as VOCs [43] depend mostly on wood species [36,50,51,52]. Naturally, the type and amount of VOCs present (and possibly released) from wood depend also on life history, interaction with biotic and abiotic factors, diseases, soil quality, nutrition, irrigation, weather and climate conditions, health of the plant, as well as its life cycle period (e.g., hibernation) at the moment of timber material production [53]. Other significant influencing factors are tree age [42], tree genetics [51], wood cut location in the log [54,55], tree growth locality [56,57], and also the impact of air pollution and fertilization [58,59]. The method of technological processing, e.g., drying before processing into final products is also important [40,60]. It is worth noticing that thermal treatment speeds up the release of terpenes from wood, and processing at higher temperatures leads to a drop of terpenes’ quantity in a final product [61]. The wood age impact on VOCs content and emission was described in the study of Ewen [30]. A decrease in the intensity of some major compounds as well as a reduction of the compounds number in the overall VOC profile was observed when comparing new seasoned pine timber and sound timber stored for approximately 100 years. Nevertheless, the widest spectrum of extractives has been observed in tropical wood species, and their content is also higher compared to wood from temperate climatic zones [35,50,62]. However, softwoods and hardwoods, especially broadleaved ones and various kinds of oaks (Quercus sp.), are more intensely industrially used than tropical woods. Conifers contain mainly resin acids, fatty acids, terpenes, and flavonoids [57,63]. There are also significant differences in their content comparing sapwood and heartwood zones [57,64], even if the composition of heartwoods’ and sapwoods’ VOCs may be very similar. Although similarities in spruce sapwood and heartwood were observed, and the same amounts of VOCs (101 compounds) were detected from sapwood and heartwood using the solid phase microextraction (SPME) technique, Z-β-ocimene occurred only in sapwood, while fenchol was present only in heartwood [63].

Benouadah et al. [65] studied the variance between heartwood and sapwood of Pinus halapensis, concluding lipophilic extractives (resin acids, terpenes, fatty alcohols) were a little more abundant in heartwood (1.6%) than in sapwood (1.1%). The content of acetic acid, in general the main volatile acid in wood, was slightly higher in sapwood than in heartwood. Nevertheless, no significant variance between heartwood and sapwood was observed in case of pines.

Valuating the most commonly used woods, some species of pine contain more extractives, compared to, e.g., common European spruce (*Picea abies*) [60]. However, the differences can be seen even in the same species. e.g., in case of European larch (*Larix decidua*) and Siberian larch (*Larix sibirica*) [43], as well as in the heartwoods extractives comparison of various larches (European larches—*Larix decidua* var. decidua, *L. decidua* var. sudetica, Japanese larches—*L. kaempferi*, *L. eurolepis*). A higher amount of phenolics in case of Japenese species strongly correlated with higher decay resistance [38]. As investigated by Forsthuber et al. [66], Siberian larch contains more extractives such as resin acids, monoterpenoids, and flavonoids than European larch, favoring this wood to be used outdoors.

Similarly, Douglas fir (*Pseudotsuga menziesii*) contains mainly resin acids, flavonoids, and tannins in heartwood, providing a good natural durability [64]. The wood of Sweet chestnut (*Castanea sativa*) and eucalyptus (*Eucalyptus* sp.) contain mainly phenols, ellagitannin [67], glycerides, and flavanols [41]. The content of phenolic components varies considerably from 1.3 to 7% depending on the tree growth location and a particular species [68]. Significant differences in VOCs content are observed, especially in case of pines [51]. Dix et al. [69] reported that in pine species, the heartwood emitted higher amounts of VOCs than sapwood. Following up, the emission of VOCs in case of pine wood can change depending on the sapwood or heartwood within the cross-section during drying. These findings are proved in the study of Sivrikaya et al. [70]; the total VOCs emissions were considerably higher in air-dried heartwood (413.16 mg m^−2^ h^−1^) than in air-dried sapwood (32.89 mg m^−2^ h^−1^) of Scots pine (*Pinus sylvestris*). Especially, among the aldehydes, hexanal and pentanal were the dominating compounds. Then, α-pinene was the major compound among the terpenes, which are a group of VOCs that typically keeps on releasing from wood at least for one year (in constant conditions) [61,71]. To demonstrate some of these findings, the most abundant VOCs emitted from different tree species, as well as concentrations of VOCs emitted from selected—commonly processed wood [72], are presented in Table 1.

Drying, either at natural conditions or driven artificially, is changing a profile of VOCs that can be emitted from wood. For example, acetic acid is formed during the drying of wood by hydrolysis of the acetyl groups of hemicelluloses [84], and furfural is formed from wood xylose in a strongly temperature-dependent reaction [85].

He, Zhang, and Wei [86] compare deciduous trees stating that hardwoods, such as oak and beech, emit primarily large amounts of acetic and formic acids and less terpenes, while hardwoods with a lower density represented by poplar (Populus tremula) emit less organic acids but more terpenes. The compounds, such as simple phenols, lignans, coumarins, or polyphenols, are also specific for oak wood [55,68,87].

## 3. VOCs from Wood-Based Panels and Products

Wooden products, especially wood-based panels, composites, and engineered wood products, became an environmental issue recently, as these are very likely the major sources of aldehydes (including formaldehyde) and terpenes in newly constructed houses [88]. Since the majority of them are used in indoor decoration and furnishing, the indoor air pollution caused by these materials may lead to the “sick building syndrome” [44,89].

In the wood-based panels industry, the trail of VOCs emission actually starts in the forests and continues ultimately into final products, where wooden fibers, particles, strands, or veneers are bonded with diverse chemical compounds and additives [44,49,90]. Then, these materials are the crucial components in the consequent furniture production where glues, adhesives, diluents, curing agents, and paints are additionally used [91]. According to the Environmental Protection Agency (EPA) [92], the sources of VOCs emissions include resins, coatings [93], and other types of finishes that can offgas and pollute indoor air [94]. An emissions test by Notheim et al. [95] determined that the overall emission rates from wood products with veneered substrates were significantly higher than the overall emission rates from wood products with melamine and vinyl substrates. This fact occurs due to the sealer and acid catalyzed topcoat used as the veneer finish. However, less harmful chemicals are being used due to environmental and health concerns, and the emissions of VOCs from additives, glues, coatings, and polymers are being steadily reduced [96]. Hence, the emission rate depends on the wood species as well as on production factors and boundary conditions, such as drying, hot pressing, storage, etc. [44]. It was shown that the VOCs emitted during wood particle drying mainly consist of terpenes [97]. Thus, terpenes are mostly derived from wood particles, not from glues and resins in wood-based panels production [79]. The study of He et al. [86] revealed that in contrast to that, urea–formaldehyde (UF) resin used for medium-density fiberboard (MDF) production had the lowest total VOCs content, while the wood chips had the highest. Comparing the glues used for MDF production, UF resin proved to have the highest emission concentration, while the melamine formaldehyde (MF) adhesive system had a lower one, and polyvinyl acetate (PVAc) had the lowest [25]. MF resin was also used in the study of Böhm et al. [81] testing formaldehyde emissions from various raw materials as well as manufactured wood. It was concluded that wood species, as well as processing, are the key factors influencing formaldehyde emission [28,86,98]. Böhm et al. [81] found six times higher formaldehyde emission from beech than from poplar, oak, or pine (84, 14, 14, and 16 µg m^−2^ h^−1^ respectively) and assumed that in processed materials, during two weeks after material manufacturing, a significant decrease in formaldehyde emission can be observed.

Liu et al. [89] emphasize the influence of processing parameters on VOCs emissions in larch particleboard (PB) production. The concentration and emission rate of VOCs were significantly affected by hot pressing temperature and time. The increase of temperature leads to an increase of total VOCs emission in the beginning. Then, the concentration of VOCs collapses dramatically within the first 60 min of heat exposure. The higher density, thickness, and resin content of larch PB were considered primary reasons leading to higher terpenes and aldehydes emissions and to a total VOCs increase. A similar trend was observed in case of press time prolonging. This phenomenon is linked to the content of wood extractives in larch. A study of Sun et al. [79] reports on the effect of larch PB density, thickness, and resin content on total VOCs and VOCs emission. Terpenes emission from the material exhibited an increment by adding density and thickness, and it dropped while increasing UF resin content. As the heat exposure time was extended during manufacture, the total VOC from all other PB samples produced under different manufacture conditions decreased.

A study from Baumann et al. [78] focused on the emissions of terpenes from PB and MDF samples from the North American production. Among the PB samples, the predominant compounds were pinenes, camphene, Δ3-carene, p-cymene, limonene, and borneol—the VOCs typical for wood (see Table 1). It was also proved that the terpenes emission from PB and MDF decreases within 4 days in a test chamber by 20 to 80%. An interesting observation was made while comparing PB and MDF produced from the same raw material. In most PB, 3-cerene and pinenes were present, while most of these compounds were absent in the MDFs. This is due to the processing of wood particles that are converted to fibers using a pulping process. The temperature in the pressurized refiner is generally held between 160 and 185 °C. This high-temperature process may drive terpenes from the material, resulting in lower emissions by the final product. According to this explanation, the terpenes with lower boiling points, such as α- and β-pinene (boiling points of 155 and 165 °C respectively), were completely absent from the MDF emissions, whereas the higher boiling terpenes, such as limonene (boiling point of 176 °C), were present only in some of the samples. Then, Liu et al. [89] presented acetic acid-butyl ester, α-pinene, and benzene as the main VOCs emitting from PB, especially after being hot-pressed. Terpenes and aldehydes are the main volatiles emitted from oriented strand boards (OSBs) [73], specifically pentanal and hexanal, which are released during the drying of hardwood flakes for OSBs. Su et al. [99] and Svedberg et al. [100] stated that these and other aldehydes are oxidation products of wood components formed during wood drying operations. The presence of hexanal is facilitated by drying at elevated temperature. The emission of hexanal lowers with time while the boards are in an air-conditioned environment [101].

A specific category of wooden products are wooden floors. The oak parquets as a frequent building material were considered risky in terms of VOCs emission, especially while being used as a top layer of flooring systems using floor heating. As described by Cecchi [16], heating may emphasize the VOCs emission. Parquet samples are expected to be VOCs emitters due to the general degradation of wood, wood volatile compounds, and volatile compounds from the coatings—as well as eventually from the adhesives used to produce a stable multilayer parquet. For example, nonanal comes from the autoxidation of the fatty acids contained in wood. Nonanal is a growth factor for wood-rotting fungi [102]. Although many aldehydes are emitted from wood flooring as a consequence of the autoxidation of fatty acids contained in wood, there is increasing evidence that the chemical reaction between ozone and terpenes such as d-limonene or alpha pinene can produce a number of different aldehydes [103]. It is worth noticing in the case of multilayer wood flooring that plywood had been mentioned as a source of α-pinene, nonanal, octanal, pentanal, and hexanal as a predominant compound [88,99,104]. The plywood subfloor, composed of softwood species, had in general comparable emissions with softwood PBs [78].

## 4. VOCs from Wood and Wood-Based Panels as a Potential Health Risk, Ways of their Mitigation

Taking into account human wellbeing, the German Committee for Health-Related Evaluation of Building Products (AgBB) [105] promotes VOCs’ effects from building materials ranging from unpleasant odors and irritation in the mucous membranes of the eyes, nose, and throat to effects on the nervous system and long-term effects. Substances causing allergy or aggravating allergic reactions and, most specifically, those with carcinogenic, mutagenic, or reprotoxic potential belong to this category. Therefore, AgBB has stated the so-called LCI values (Lowest Concentration of Interest) for 184 compounds such as terpenes and aldehydes that usually occur in building materials concerning wood-based panels. Setting up the limit values might secure a low VOCs emission materials production.

Various terpenes—alpha-pinene, beta-pinene, and hexanal—are considered irritating to eyes, respiratory system, and skin [16]. Decanal and nonanal cause irritation to eyes and skin, while furfural irritates eyes and skin and is noted for limited evidence of a carcinogenic effect. Alpha-pinene may be harmful by inhalation and in contact with skin. According to Mølhave [106], concentrations of VOCs up to 25,000 µg m^−3^ lead to headaches and other neurotic (derogative for the nervous system) symptoms. Formaldehyde can cause eye and upper respiratory tract irritation, and moreover, it was classified as a Group 1 human carcinogen by the International Agency for Research on Cancer [13]. On the other hand, Gminski et al. [107] tested the impact of pine wood and OSBs VOCs’ emission on human sensory irritations and found no adverse effects on the eyes, nose, throat, upper airways, or lung function after exposure to even the highest VOC levels (concentrations of up to 13,000 µg m^−3^). Eye blink frequency as a parameter for irritation was not affected during or after exposure. Sensorial perception of odor was the only detectable effect—odor of both pine wood and OSB was considered as more “pleasant” than “unpleasant”. Moreover, the study from the Institute of Health Technology and Prevention Research [108] proclaims the positive effect of Stone Pine (Pinus cembra) essential oils from furniture and cladding on human health in terms of stress inhibition, breath soothing, and heart frequency reduction leading to relaxed feelings.

Since wood VOCs’ presence in the indoor air is a case of concern, ways to reduce VOCs release from wood are still in demand. McDonald and Wastney [109] described the effect of thermal treatment on solid wood VOCs emission, showing an increase of about 60% at 140 °C compared to 120 °C. These findings were proven by Kačík et al. [61]. The thermal modification at the temperature of 60 °C accelerates the terpene emission and at the temperature 120 °C removes the terpenes almost completely. Heat treatment of spruce and pine wood significantly reduces VOCs emission and at the same time changes their composition compared to untreated or naturally air-dried wood. In particular, terpene emissions in case of spruce and pine decrease during the heat treatment process. Concerning both conifers and poplar, heat treatment leads to a reduction in hexanal emissions but evokes an increase in furfural emissions for both conifers and deciduous trees. Nevertheless, the thermal treatment can be used as a suitable method for VOCs emission mitigation, leading to a reduction of a potential health risk caused due to humans’ exposure to VOCs. The heat treatment of wood makes wood a suitable and harmless material for use in indoor environments [71].

In case of wood-based materials, manufacturing parameters optimization, mainly regarding temperature and press time, reduce VOCs emission [86]. Jiang et al. [110] showed that the heat treatment of PB (at 50 or 60 °C) reduced formaldehyde and other volatiles emissions significantly. Prolonging the bake-out time and increasing the temperature provides material that tends to emit less volatiles when back at room temperature. Nevertheless, optimal conditions should be selected for different PB to avoid material damage.

The application of coatings containing dispersed nanoparticles may lead to a total VOCs emission reduction of up to 38.6% [108]. Meanwhile, the application of cashew nut shell liquid resin for the maple face of veneer bonding on plywood [111] or even adding scavengers, such as pozzolan, directly into the medium-density fiberboard (MDF) formulation, lead to a total VOCs emission decrease. Enhanced air exchange in a ventilated chamber that simulates room conditions leads to a decrease in VOCs concentrations [110,112].

Alternative processing and raw materials for PB production are being tested with the aim to produce more environmental friendly construction materials. Omitting glues in fiberboards and the use of various renewable materials seems to be promising [113,114]. Simon et al. [115]. demonstrated that waste from coriander production can serve as a low-emission raw material for PB production. In the case of formaldehyde, 300–600 times less was emitted compared to wood MDF and particle board. Adamová et al. [116] compared VOCs from spruce chips and differently treated Cannabis sattiva shives, showing lower overall emissions from an alternative material.

## 5. Analytical Methods to Assess VOCs

Regarding the instrumental analytical methods used to determine the VOCs, gas chromatography coupled to mass spectrometry is most often used for separation and detection. The foregoing steps—volatiles extraction and sample introduction—depend strongly on the aim of the analysis. For solid sample description as a means of compounds content, exhaustive extraction techniques take place. Usually, these comprise solid–liquid extraction, which is often assisted by heat or sonication and followed by liquid injection into GC-MS. In the case of volatiles emitted from the sample, plain headspace air sampling, or more often, equilibrium techniques are used, followed by the thermal desorption of collected compounds into the analytical system. This approach is often used for indoor air monitoring or emission rates of compounds from various materials [44,61,63,70,74,85,117,118,119]. For examples of the different analytical approaches and techniques used, see Table 2.

### 5.1. GC-MS for VOCs Detection from Wood and Wood-Based Panels

Gas chromatography (GC) is today the most important analytical method in organic chemical analysis for the determination of individual low molecular substances in complex mixtures. For compounds detection, conventional flame ionization detector (FID) can be used. However, mass spectrometry (MS) is a universal and sensitive detection method, providing data for both the identification of compounds based on their mass spectra and also for their quantification when providing both quantification and confirmation ions in one run [120].

A suitable GC capillary column needs to be selected for the separation of analytes in the sample—the most often used types are nonpolar columns (−5% or 1% modified polydimethylsiloxane) or polar wax columns (Table 2). According to ISO 16000-6 [121], columns of a length of 30 m are common, which are characterized by an internal diameter of 0.25 to 0.32 mm and phase thickness of 0.25 to 0.5 µm.

Comprehensive two-dimensional gas chromatography (GC × GC) is allowing better sensitivity due to a combination of two columns, usually of a different polarity, and a modulation step, where an eluate from a first column is cryo-focused before injection onto a second column. This way, coelutions appearing in single dimension analysis can be resolved, and matrix components can be separated from target compounds. Longer columns can be used for the same purpose but unavoidably prolonging the total run time [42,62,116,122,123,124,125].

For basic measurements, a widely used quadrupole mass spectral analyzer is sufficient. However, advanced analyzers such as time of flight (TOF; either unit or high resolution) can offer beneficial properties in case of the nontarget type of analysis. Combined instruments coupling either quadrupole and TOF or multiple quadrupoles can increase the sensitivity of determination. A higher resolving power of detection can increase samples‘ throughput, since for chromatographic separation, a faster ramping can be used [125].

In mass spectrometric detection, electron impact ionization is used as a first-choice option, since the initial identification of chemical compounds can be based on mass spectral similarity with the in-built mass libraries (NIST, Wiley) or various online sources. For confirmation of target compounds identity, retention times of respective standards could be used, or calculated Kovats retention indices (KI) may be compared with literature data [116,118,123,126,127]. The amount of compounds present in the solid material or emitted to the air can be expressed exactly using calibration curves or as an equivalent of one compound (e.g., toluene) [71]. For comparison, peak areas in the total ion current (TIC) chromatogram or sum of peak areas can be used [30,70].

### 5.2. VOCs Extraction Techniques and Sample Introduction

#### 5.2.1. Liquid Extractions from a Solid Sample

Leaching or solid–liquid extraction is the process of solute component removal from the solid sample by using a liquid solvent. The methods most often used are Soxhlet extraction [130], hydrodistillation, and maceration. The latter named method can be assisted by shaking or ultrasonication. The advantage of ultrasound waves lies in the penetration ability of the matrix material while rupturing the cell walls and driving the solvent into the matrix to extract the target components [132,133].

Solvents frequently used are n-hexane, alcohols (ethanol, methanol), or other solvents such as acetone or dicholoromethane. Based on the aim of a study, mixtures of solvents are used either to improve extraction yield or to simulate a specific solvent (water/ethanol) in case of VOCs extraction from casks or wood chips to various alcoholic beverages. Naturally, the extraction power of different solvents should be taken into account when designing the method for a target group of compounds.

The Soxhlet apparatus has been used in a number of studies for the extraction of various sample components, including volatile and semivolatile compounds [52,61,65,89]. In principle, a repeated extraction of a solid sample is performed with condensed vapor of hot solvent in a glass apparatus. When the extraction chamber is full, then it is automatically emptied using siphon. The extracted compounds are being concentrated in a distillation flask below the extraction chamber. In the last decade, focusing on costs reduction and more environmental-friendly extraction, alternative approaches to traditional Soxhlet apparatus were introduced. A similar principle is used in the Soxtec instrument (repeated automated extraction by solvent) or the PLE (pressurized liquid extraction), which is also called ASE (accelerated solvent extraction) [134]. Based on the comparison with the traditional Soxhlet apparatus, PLE is considered as a greener option, since it has similar efficiency, is faster, and uses lower amounts of organic solvents [135,136,137,138].

Hydrodistillation is often used for essential oils extraction from various plant materials, including wood. It is also suitable for the extraction of semivolatiles’ constituents. Three hydrodistillation methods are considered: (i) direct water distillation, when the material is boiled with water in a flask and a mixture of extracted compounds, and water steam is cooled down, and collected; (ii) more gentle, water–steam extraction, where the material is exposed to steam from boiling water below, preventing extracted material from making contact with the bottom of the extraction flask where overheating can occur; and (iii) direct steam extraction, when steam is generated outside of the extraction vessel, reducing the extraction time significantly [63,135,139,140,141,142]. In hydrodistillation, the extracted material is exposed to temperatures close to 100 °C, which can cause the degradation of thermolabile compounds. In case of boiling with water, also an unwanted reaction between extracted compounds can take place.

Depending on the matrix extracted, authors comparing organic solvent extractions with hydrodistillation reported similar qualitative information, while for specific compounds, the quantitative yield was better in the case of organic solvent extraction [63,122].

#### 5.2.2. VOCs Sampling from Air

##### Headspace

Headspace (HS) sampling is an easy way of volatile compounds collection, taking the defined volume of the air above the solid (e.g., indoor air with various furniture, air from test chamber) to be injected into GC-MS. The equilibrium between the compounds’ amount present in a solid material and compounds’ vapors in the headspace area is affected (aside from the sample form itself) mostly by temperature. Elevating the temperature can be used to enhance VOCs emission, thus enhancing the sensitivity of a measurement. Nevertheless, since no concentration step is employed in the procedure, this approach is less sensitive than other discussed air sampling techniques. On the other hand, due to the vacation of sorbent, no discrimination of compounds, based on different affinity to the sorbent is taking place [83,120,143,144].

##### Sorption Techniques Coupled to Thermal Desorption

Various experiments focused on VOCs emission were carried out using different combinations of sorption from a headspace and thermal desorption into GC. A standardized method defined in an International standard ISO 16000-6 [121] had been developed for the determination of volatile organic compounds in indoor air. For this purpose, air in the test chamber (made from stainless-steel or glass) is sampled for volatiles using a calibrated pump and flow meter [145]. A predetermined volume of air is drawn through sorbent-filled tubes (usually Tenax TA^®^), where the adsorption of compounds in the range n-C_7_ to n-C_30_ takes place [120]. A sample of a material, e.g., PB or solid wood, is placed in the chamber, and the sampling is performed following defined time intervals (on day 1, 3, 7, 14, 28, eventually 56) [121]. For an identical purpose, Tenax GR was used by some authors. Then, desorption temperatures depend on the sorbent type used and on compounds expected to be collected on the sorbent [30,71]. In addition, the desorption flow rate and time can vary, but they always have to ensure sufficient sample transfer from the sorption device to a GC inlet, while avoiding losses of volatile compounds [70]. Then, a cryofocusing unit is an important component for the cooling of an inlet of GC or the first part of the column to condensate compounds eluted from a sampling tube in the thermal desorption unit [120]. Peltier effect coller, liquid CO_2_, or nitrogen are usually used for cooling. After the cryofocusing period is terminated, volatiles are separated and detected using GC equipped with various detectors [70,74].

A disadvantage of the ISO 16000 approach lies in a long time delay until the sample is in a measurable state. Nevertheless, different modifications of the ISO 16000 approach were presented—either in case of different test chamber volumes or in various combinations of sorbent or time of sample preparation or volatiles sampling (Table 2). Portable cells (e.g., DOSEC or FLEC) combined with GC-MS are allowing almost online measurements of VOCs, including formaldehyde, emission from a material in situ [98,146].

Solid wood samples also may be subjected to thermal treatment directly in the thermal desorption (DTD) glass tube of thermo-desorber, and the volatiles formed may be analyzed by GC-MS [30,131,147].

##### SPME

Solid phase microextraction (SPME) is a sensitive, fast, and solvent-free analyte extraction technique including preconcentration and sample introduction invented by prof. Pawliszyn in the late 1980s [148]. The SPME unit consists of a fused silica fiber coated with a more or less selective stationary phase. The most often used commercially available fiber stationary phase is adsorptive divinylbenzen/carboxen/polydimethylsiloxane (DVB/CAR/PDMS) for a wide range of sampled compounds polarity, or absorption phases e.g., polydimethylsiloxane (PDMS) and polyacrylate (PA) for non-polar and for more polar compounds, respectively [16,30,43,63,76,148].

With the exception of wood extracted volatiles into water or water/ethanol simulating solvent, SPME in wood volatiles analysis is usually performed from headspace. In an above-mentioned case, the direct immersion of fiber into the liquid can be more sensitive than the sorption from the headspace above, since only a partition between the fibers´ stationary phase and volatiles extracted in liquid takes place [128]. In the HS option, a partition between solid/liquid extract and headspace air must take place also.

Comparing SPME with another extraction technique used for wood sample description, it was proved that this approach can be as sensitive as water distillation for highly volatile compounds while requiring less sample material and allowing the automated analysis of a large number of samples. The use of this approach for semivolatile compounds is of course limited [16,75,76,77,122,149].

## 6. Conclusions

Wood and wood-based materials contain a large number of different volatile organic compounds (VOCs) that may affect the quality of the indoor air (indoor environment) in humans’ living/work spaces. The review provides an overview of VOCs contained in native wood as well as comments on additives used in wood-based panels’ production. The VOCs content in wood is influenced mainly by the wood species, the proportion of heartwood and sapwood, the tree age, the locality of tree growth, and the subsequent technological process while wood processing, especially by drying. Other important factors arise in wood-based panels’ production. In particular, these include the type of composite material, the binder used (glue), the specific production technology used, the proportion and type of other additives, and the final surface treatment. The variability in the total amount of compounds detected can also be strongly affected by the analytical method used. Therefore, the review also describes the results of previous studies and various analytical methods used to determine the VOCs released from wood and wood-based panels.

The most often applied analytical approaches use various volatile compounds collection followed by gas chromatographic separation coupled to mass spectrometric detection. Volatile compounds collection from air was mostly performed using the sorption principle, employing sorbent tubes or SPME fibers. However, information on the extractable volatiles present in solid samples is also important. For this reason, approaches for the extraction of less volatile compounds from solid materials are introduced. Contrary to the application of conventional flame ionization detection, mass spectrometric detection allows compounds identification based on a comparison of obtained spectra with spectra in spectral libraries, which is beneficial in case of a nontarget type of analysis.

The review provides a brief guidance on how to reduce the potential health risks arising from excessive concentrations of harmful substances released into the interiors and also an overview of techniques often used for wood volatiles analysis.

## Figures and Tables

**Table 1:** VOCs emitted from wood.

Extractives/Group of VOCs	VOC	Pine *		Spruce *		Larch *		Fir	Douglas Fir	Aspen	Oak	Beech	Wood Species
		Concentration in the Test Chamber (µg/m^3^) Sapwood/Heartwood		Concentration in the Test Chamber (µg/m^3^) Sapwood/Heartwood		Concentration in the Test Chamber (µg/m^3^) Sapwood/Heartwood							
**Terpenes**	α-pinene	**3459/294**	[31,44,60,70,71,73,74]	**119/320**	[60,71,75]	**126/509**	[76]	[61,77]	[78]				**Reference**
	β-pinene	**13/16**	[44,60,70,74]	**−/74**	[60,71,75]	**4/14**		[61,77]	[78]				
	Camphene	**23/10**	[44,70,71,74]	**<1/−**	[71,75]	**<1/6**	[76]	[61,77]					
	Δ3-carene	**108/40**	[31,44,60,70,71,74]	**63/45**	[71,75]	**17/16**	[76]	[61,77]	[78]				
	Limonene	**5/<1**	[31,44,70,74]	**30/19**	[60,71,75]	**13/7**		[61,77]	[78]				
**Aldehydes**	Benzaldehyde	**<1/6**	[70,71,74]	**<1/1**	[75]	**7/3**	[79]			[71]			
	Decanal	**11/16**	[70,74]	**−**	[71,75]	**7/−**				[71]			
	Furfural	**/**	[70,71,74]	**/**	[71]	**/**				[71]			
	Hexanal	**4/162**	[31,44,70,71,74]	**−/17**	[71,75]	**8/24**	[76,79]			[71]	[16]		
	Nonanal	**4/12**	[71,74]	**4/12**	[71,75]	**7/10**	[76,79]			[71]			
	Octanal	**1/7**	[70,74]	**<1/−**		**5/5**				[71]			
	Pentanal	**−**	[70,71,74]	**−**		**−/18**				[71]			
	Formaldehyde	**/**	[80,81]	**/**	[80,81]	**/**				[81]	[81,82]	[81]	
**Acids**	Acetic acid	**/**	[31,71,74]	**/**	[71]	**/**	[76,79]			[71]	[16,83]	[83]	

Note: a group of most abundant VOCs emitted from different species of wood, comprising concentrations of VOCs emitted from sapwood/heartwood on day 31 -values based on the recent study from Czajka et al. [72]. Some other compounds, e.g., Thymol, Myrtenal, Thujen, Terpinen or Terpineol, were detected by GC-MS. * most often used industrial wood species.

**Table 2:** Methods applied to assess VOCs from wood and wood-based panels.

Material	Aim	Analytical Method	Sample Extraction and Introduction Technique	Capillary Column (Length × Internal Diameter; Film Thickness)	Ref.
***Larix sibirica* vs. *Larix decidua***	variability in VOCs composition, VOCs intensity	GC-FID, GC-MS	SMPE: DVB-CAR-PDMS—50:30 μm	SLB-5 (30 m × 0.25 mm; 0.25 μm)	[43]
***Picea abies***	variability in VOCs composition, methods comparison	GC-MS	SPME: DVB-CAR-PDMS—50:30 μm; CAR-PDMS—75 μm; CW-DVB—70 μm; PDMS-DVB—65 μm/dynamic HS/hydrodistillation	HP-5 (30 m × 0.32 mm; 0.25 μm)	[63]
***Larix gmelinii***	variability in VOCs composition, methods comparison	GC-MS	SPME: PDMS—100 μm/static headspace	TR-V1 (30 m × 0.25 mm; 1.4 μm)	[76]
***Serpula lacrymans*** **,** ***Coniophora puteana* and *Pinus sylvestris***	variability in VOCs composition, methods comparison	GC-MS	SPME: PDMS—100 μm; polyacrylate—85 μm, Tenax GR tubes	HP-1, HP-5, HP-Innowax (30 m × 0.25 mm; 0.25 µm)	[31]
**unspecified wood biomass**	furfural extraction and identification	GC-MS	autohydrolysis; SPME: DVB-CAR-PDMS; *	HP-5 MS (30 m × 0.25 mm; 0.25 μm)	[128]
**wooden parquets**	variability in VOCs composition	GC-MS	SPME: DVB-CAR-PDMS—50:30 μm	HP-5MS (30 m × 0.25 mm; 0.25 μm)	[16]
***Abies alba* vs. *Fagus sylvatica***	methods comparison due to VOCs	GC-MS	glass TD tube with glass wool and TD	DB-5 (30 m × 0.25 mm; 0.25 μm)	[129]
***Larix gmelinii***	TVOC and VOCs quantification (µg m^−3^)	GC-MS	glass desiccator (0.015 m^3^) and Tenax TA© tubes	TR-V1 (30 m × 0.25 mm; 1.4 μm)	[79]
***Picea abies*, *Pinus sylvestris*** **vs. *Populus tremula***	TVOC comparison	TCT-GC-MS	metal chamber (0.12 m^3^) and Tenax GR	HP-5MS (50 m × *; 0.5 μm)	[71]
***Pinus sylvestris***	variability in VOCs, quantification	GC-MS	FLEC (0.00035 m^3^) and Tenax TA© tubes	*	[70]
***Pinus sylvestris***	TVOC, relative proportion (% of total emission) of different compound groups and individual compounds	GC-MS	glass container (0.015 m^3^) and Tenax TA© tubes	HP-5 (50 m × 0.2 mm; 0.5 μm)	[74]
**MDF**	TVOC emission rate (mg m^−2^ h^−1^)	GC-MS	chamber (0.020 m^3^) and Tenax TA© tubes	RTX-1 (105 m × 0.32 mm; 3 µm)	[112]
**PB and MDF** **from various tree kinds**	VOCs quantification	GC-MS	stainless-steel chamber (0.053 m^3^) and cryotrap	EC-5 (30 m × 0.25 mm; 25 μm)	[78]
**organic vs. unorganic insulation**	TVOC	GC-MS	stainless-steel chamber (0.58 m^3^) and Tenax TA© tubes	fused silica column (25 m × 0.32 mm; *)	[33]
**OSB from *Pinus sylvestris***	aldehydes and terpenes—chambers comparison	GC-MS	glass desiccator (0.023 m^3^) and stainless-steel chamber (1 m^3^) and Tenax TA© tubes, TDS 3	*	[73]
**OSB**	individual VOCs quantification	GC-MS	glass desiccator and Tenax TA© tubes	*	[101]
**Coatings in a furniture workshop**	variability in VOCs composition, quantification	GC-MS	Tenax TA© tubes	DA-WAX (30 m × 0.25 m; 0.25 μm)	[93]
***Pinus silvestris* vs. *Picea abies***	abundance of monoterpenes	GC-MS	Tenax TA© tubes—acetone and Soxtec©	DB-Wax (30 m × 0.25 mm; 0.25 μm)	[60]
**12 various tropical wood species**	total amount of extractives (% to dry wood)	GC-MS	sodium hydroxide and Soxhlet	HP-1 (25 m × 0.2 mm; 0.11 μm)	[62]
***Populus cathayana*** **vs. *Hevea brasiliensis***	individual VOCs%	GC-MS/O	ethanol and toluene and Soxhlet	DB-Wax (30 m × 0.25 mm; 0.25 μm)	[39]
***Larix gmelinii* PB**	individual VOCs%	GC-MS	methylene chlorid and Soxhlet	*	[77]
***Picea abies* vs. *Abies alba***	individual VOCs quantification, methods comparison	GC-FID, GC-MS	ASE vs. steam distillation vs. Soxhlet	DB-5 (30 m × *; *)	[130]
***Abies alba* Mill.**	VOCs reduction as protection from wood decay	GC-MS	extraction by hexane in Promax 2020 shaker	HP-5 MS (30 m × 0.25 mm; 0.25 μm)	[61]
***Quercus alba*, *Quercus robur*** **vs. *Quercus pedunculata***	specific VOCs quantification (cis- and trans-ß-methyl-γ-octalactone, eugenol, vanillin and syringaldehyde)	(DTD)-GC-MS	extraction by dichlormethane	SPB-1 (50 m × 0.2 mm; 0.25 μm)	[131]
**Construction materials**	VOCs emission from construction material	GC-(FID)-MS	DOSEC-SPME	*	[98]

* value unspecified; Abbreviations.: TVOC—total volatile organic compounds; TD—thermal desorption; DTD—direct thermal desorption; TCT–thermal-desorption cryo-trapping; FID—flame ionization detection, GC-MS/O—GC-MS/Olfactometry, FLEC—field and laboratory emission cell, DOSEC—device for on-site emission control.
